# Dementia Caregiver Surrogate Decision Making Self-Efficacy:Distress, and Quality of Life

**DOI:** 10.1093/geroni/igab046.2756

**Published:** 2021-12-17

**Authors:** Giovanna Pilonieta, David Geldmacher

**Affiliations:** 1 University of Alabama at Birmingham, Birmingham, Alabama, United States; 2 University of Alabama at Birmingham, University of Alabama at Birmingham, Alabama, United States

## Abstract

We assessed the relationship between caregiver self-efficacy and caregivers’ ratings of care recipient’s health-related quality of life, the severity of neuropsychiatric symptoms, and associated caregiver distress for persons with Alzheimer’s dementia (AD). Methods: The 31-item DEMQOL-Proxy, Neuropsychiatric Inventory (NPI-Q), and the Self-Efficacy for Surrogate Decision-Making scale (SDM-SES) were collected from 26 family caregivers of people with AD expressing care resistant behaviors. We used Spearman correlations to assess relationships between SDM-SES, NPI-Q severity, and NPI-distress and DEMQOL-proxy. Among enrolled caregivers, 14 (54%) were women; mean age was 64.5 years, and 24 (92%) were college-educated. Their care recipients were 61% women, 77 % white, with a mean age of 76 years, and mostly college-educated (88%). Mean scores were DEMQOL-Proxy 91.27 (+/- 14.16), SDM-SES 16.38 (+/- 2.74), NPI-Q Severity score 14.23 (+/- 6.04), and NPI-distress 17.42 (+/-6.90). There were moderate correlations between DEMQOL-Proxy and SDM-SES (r=0.54), NPI severity (r= -0.42) and NPI-distress (r= -0.49). Secondary analysis showed a moderate correlation between SDM-SES and NPI-distress (r= -0.40). We identified associations between caregiver self-efficacy, quality of life, and caregiver distress. A higher baseline SDM-SES was associated with greater health-related quality of life for the care recipient. Lower self-efficacy scores were related to more caregiver distress related to neuropsychiatric symptoms. Higher NPI severity and caregiver distress were associated with lower quality of life for the care recipient. Interventions targeting self-efficacy may promote improved QOL and decrease caregiver distress in AD dyads.

